# Efficacy of the Combination Therapy of Intense Pulsed Light and Microneedling With a Postbiotic Formulation for Melasma

**DOI:** 10.1111/jocd.70580

**Published:** 2025-12-05

**Authors:** Zhanhong Li, Yiyuan Xiang, Juan Meng, Huiying Wang, Hao Wu, Ting Li, Xiaoling Jiang, Tao Wu, Yan Zhang, Xuehua Xiao, Xiufen Ma, Mei Yang, Han Zhu, Yan Bi, Yurong Chen, Hong Lei, Jing Hu, Guiyun Huang, Lin Cong, Jing Wang, Lei Yan, Han Zhou, Chunqing Li, Xue Zhang, Mei Ou, Qiong Meng

**Affiliations:** ^1^ Guangzhou MLT Medical Cosmetic Clinic Guangzhou China; ^2^ Department of Cosmetic Dermatology Sichuan Huamei Zixin Plastic Surgery Hospital Chengdu China; ^3^ Department of Cosmetic Dermatology Foshan Huamei Liancheng Plastic Surgery Hospital Foshan China; ^4^ Department of Cosmetic Dermatology Changsha MYLIKE Medical Cosmetic Hospital Changsha China; ^5^ Medical Cosmetic Center Xinjiang Uiger Municipal People's Hospital Urumqi China; ^6^ Fuzhou Gulou Aimeer Medical Cosmetic Clinic Fuzhou China; ^7^ Department of Cosmetic Dermatology Hainan Huamei Medical Cosmetic Hospital Haikou China; ^8^ Department of Cosmetic Dermatology Xi'an Yezi Medical Cosmetic Hospital Xi'an China; ^9^ Fuqing Yinxi Chen Yilei Medical Cosmetic Clinic Fuqing China; ^10^ Department of Cosmetic Dermatology Xiamen MYLIKE Medical Cosmetic Hospital Xiamen China; ^11^ Center for Photomedicine Chengdu Runhe Dermatology Specialist Hospital Chengdu China; ^12^ Department of Cosmetic Dermatology Hangzhou MYLIKE Medical Cosmetic Hospital Hangzhou China; ^13^ Department of Cosmetic Dermatology Xinjiang Huamei Plastic Surgery Hospital Urumqi China; ^14^ Department of Cosmetic Dermatology Jinan Jingmei Boge Medical Cosmetic Hospital Jinan China; ^15^ Department of Cosmetic Dermatology Guangzhou MYLIKE Medical Cosmetic Hospital Guangzhou China; ^16^ Department of Cosmetic Dermatology Shenzhen Yestar Medical Cosmetic Hospital Shenzhen China; ^17^ Department of Cosmetic Dermatology Shenzhen Pengai Medical Cosmetic Hospital Shenzhen China; ^18^ Chengdu Gaoxin Preface Medical Cosmetic Clinic Chengdu China; ^19^ Guangzhou LTHINK Medical Biotechnology Co. Ltd Guangzhou China; ^20^ Department of Cosmetic Dermatology Yunnan Wushi Jiamei Medical Cosmetic Hospital Kunming China; ^21^ Department of Cosmetic Dermatology Xi'an Gao Yi Sheng Medical Cosmetic Hospital Xi'an China; ^22^ Department of Cosmetic Dermatology Sichuan Hamilton Medical Cosmetic Hospital Chengdu China

**Keywords:** intense pulsed light, melasma, mesotherapy, microneedling, postbiotics

## Abstract

**Background:**

Melasma pathogenesis involves multifactorial mechanisms, and a comprehensive strategy achieves superior outcomes compared to monotherapy. Intense pulsed light (IPL) reduces pigmentation via selective photothermal effects, while microneedling enhances melanin clearance through mechanical stimulation and improves transdermal drug delivery. However, the therapeutic synergy of combining IPL with microneedling for melasma management requires further validation.

**Objective:**

To assess the clinical effectiveness of combining IPL and microneedling with a postbiotic formulation containing Lactobacillus fermentation lysate in facial melasma.

**Methods:**

Thirty individuals were enlisted and randomly split into two groups. IPL therapy group: Received IPL treatments at 4‐week intervals for 12 weeks. Combination therapy group: Underwent alternating IPL and microneedling every 2 weeks for 12 weeks. Symptomatic improvement was assessed utilizing the Modified Melasma Area and Severity Index (mMASI) Score and VISIA skin detection system (Canfield, USA). Patient satisfaction was evaluated through questionnaires.

**Results:**

Both groups showed significant mMASI score reductions from baseline, with greater improvement in the combination group (*p* < 0.05). Clinical photographs demonstrated decreased pigmentation and inflammation, with 73.3% of patients reporting satisfaction. VISIA analysis confirmed the significant decrease in the score of speckle, brown speckle, red region and UV speckle, further confirming the positive impact of the combination therapy on melasma. No severe adverse events occurred.

**Conclusion:**

The combination therapy of IPL and microneedling with a postbiotic formulation exhibits a multidimensional synergistic effect, presenting a promising new direction for melasma therapy.

## 1 Introduction

Melasma, characterized by hyperpigmented areas, clinically manifests as irregular brown spots on the face that are typically symmetrically distributed and vary in color intensity [1]. This condition not only has a detrimental impact on the patient's facial aesthetics but also adversely affects their self‐esteem and overall quality of life. The rate of melasma ranges from 8.8% to 40% across different ethnic groups, predominantly affecting individuals with Fitzpatrick skin types III to V, and shows a marked female predilection during reproductive years [2, 3]. The onset of melasma is linked to a multifaceted interplay of factors, including sunlight exposure, hormonal fluctuations, and genetic predisposition [4, 5, 6]. Key mechanisms include upregulated melanogenesis, abnormal vascular proliferation, chronic inflammation, and impaired skin barrier function. Histologically, melasma is categorized into epidermal, dermal, and mixed subtypes, determined by the depth of pigment deposition [7]. Current therapeutic options include topical depigmenting agents (e.g., hydroquinone), laser therapies, and phototherapy [8]. Nonetheless, achieving optimal therapeutic outcomes continues to present considerable challenges in clinical practice.

Intense pulsed light (IPL) is characterized as a high‐intensity, broad‐spectrum light that is emitted in a pulsed sequence, with wavelengths spanning from 400 to 1200 nm. Its efficacy in melasma treatment primarily relies on selective photothermolysis [9]. Through optical filters and adjustable pulse parameters, light energy is directed to target chromophores at specific depths. In the targeted lesion areas, melanin efficiently captures these photonic energies and transduces them into thermal energy, resulting in the breakdown and disintegration of melanin particles [10]. Concurrently, the resultant thermal energy dissipates into adjacent tissues, stimulating accelerated differentiation of epidermal keratinocytes. During this process, as keratinocytes migrate superficially, melanosomes undergo gradual segregation, thereby reducing epidermal pigmentation load [11].

Microneedling is designed to produce micro‐traumas in a controlled manner, in order to activate the self‐repair and regeneration mechanisms of skin [12]. In addition, microneedling creates transient microchannels, significantly enhancing the absorption of medications or active ingredients [13]. In melasma management, the procedure is strategically combined with targeted products aimed at restoring the skin barrier and reducing melanin production [14]. Recent research suggests that immunohistochemical analysis of patients with melasma who have received microneedling treatment reveals several significant improvements, including an increase in epidermal thickness, a reduction in melanin deposits, an enhancement in collagen density within the upper dermis, and the restoration of the basement membrane bands [15, 16].

Emerging research suggests that microorganisms residing on the skin surface play a crucial role in maintaining the structural integrity of the skin barrier, supporting immune system functions, and providing defense against external pathogenic bacteria [17]. Additionally, these microorganisms contribute to various physiological processes, including skin metabolism, tissue regeneration, and the modulation of inflammatory responses [18]. In recent years, postbiotics have garnered significant attention as an emerging therapeutic modality. Postbiotics primarily refer to microbial metabolic products generated through fermentation, which exhibit various biological activities including immune regulation, anti‐inflammatory effects, and antioxidant properties [19]. Lactobacillus, a naturally occurring probiotic in the human body, has a high safety profile, and its fermentation products typically have no toxic side effects [20]. Therefore, Lactobacillus fermentation lysate is milder than certain chemical drugs or topical products in skin care or treatment processes. Researchers have found that lactic acid and short‐chain fatty acids produced by Lactobacillus during fermentation can help regulate the acid–base balance of the skin microenvironment by lowering the skin's pH value, inhibiting the growth of harmful pathogens, and promoting the restoration of skin barrier function [21].

Considering the complementary mechanisms of action of IPL and microneedling, their combined use is expected to enhance therapeutic efficacy. The addition of a subsequent postbiotic formulation is expected to enhance the effectiveness and promote accelerated repair of wounds. In this study, we aim to explore the novel therapeutic synergy achieved by integrating EVE‐CHARM whitening lotion, a postbiotic formulation, with IPL and microneedling in the treatment of facial melasma, offering a more holistic and multifaceted treatment option for melasma.

## 2 Materials and Methods

### 2.1 Study Design

This clinical trial was designed to assess the efficacy and safety of the combination of IPL and microneedling with EVE‐CHARM whitening lotion, versus IPL monotherapy in melasma management. From March 2024 to October 2024, a cohort of 30 eligible patients diagnosed with melasma was systematically recruited for participation in the study conducted in Guangzhou MLT Medical Cosmetic Clinic. The ethics committee of Guangzhou MLT Medical Cosmetic Clinic approved the study (No. 2024001). Following being thoroughly informed about the study procedures and potential risks, all participants gave their written consent. This was a single‐blind randomized clinical trial. Patients were allocated to the IPL monotherapy group or the combination therapy group using block randomization. While patients were aware of their treatment due to the nature of the procedures, outcome assessors were blinded to treatment allocation to minimize assessment bias.

### 2.2 Inclusion and Exclusion Criteria

Participants eligible for this study are those diagnosed with melasma and aged 30 to 55 years, who demonstrated overall good health as determined by comprehensive physical assessments and laboratory evaluations, and exhibited facial symmetry within the treatment region. Individuals who have undergone facial cosmetic procedures or treatments—such as laser treatments, radiofrequency treatments, or injectable therapies—within six months before, during, or after the predetermined treatment timeline are not eligible for participation. Additionally, subjects using pharmacological agents with potential effects on the study outcomes (such as anticoagulants, phenylphenols, and other phototoxic agents) were deemed ineligible for participation. The study also excluded female participants with concurrent congenital disorders, individuals experiencing facial infections, patients with coagulation disorders, those exhibiting photosensitivity, as well as pregnant and lactating individuals.

### 2.3 Materials

#### 2.3.1 IPL Device

IPL treatment in this study used the M22 aesthetic treatment system (China National Medical Products Administration Approval No. 20173247065, Lumenis Co. Ltd., Yokneam, Israel).

#### 2.3.2 Microneedling Instrument

The microneedling instrument (Guangdong Food and Drug Administration Approval No. 20222200879, Guangzhou LTHINK Biotechnology Co. Ltd., China) featured 192 sterile needles (0.22 mm diameter × 0.5 mm length) per disposable roller unit.

#### 2.3.3 The Postbiotic Formulation

The EVE‐CHARM whitening lotion, is a marketed postbiotic formulation (Guangdong Cosmetics Approval No. 20231243, Guangzhou LTHINK Medical Biotechnology Co. Ltd., Guangzhou, China). Product details can be found in the Supporting Information. Follow the instructions, thoroughly mix the lyophilized powder with the matched solution before use (final pH 6.8), and the stabilized emulsion was topically administered within 5 min post‐mixing (component detailed in Table 1).

**TABLE 1:** List of main ingredients in EVE‐CHARM whitening lotion.

	The lyophilized powder	The matched solution
Main ingredients	Water, mannitol, Lactobacillus fermentation lysate, trehalose, nicotinamide, tranexamic acid	Water, glycerol, 1,2‐hexanediol, p‐hydroxyacetophenone, allantoin, PEG‐40 hydrogenated castor oil, panthenol, sodium hyaluronate

### 2.4 Treatment

Following comprehensive education on therapeutic protocols and recovery management, participants executed protocol‐specific consent documentation confirming their understanding of both clinical procedures and research participation. Subjects were randomly divided into either the IPL therapy group or the combination therapy group, with equal numbers allocated via block randomization, and the treatment area was thoroughly disinfected before surgery. Patients of the IPL therapy group were treated with the IPL device (M22 aesthetic treatment system). The treatment parameters were set as follows: a wavelength of 590–640 nm, pulse width between 3.0 and 4.0 ms, a pulse delay time of 40–50 ms, and an energy density of 14–18 J/cm^2^. During treatment, the energy density was adjusted according to the patient's tolerance and immediate response. The treatment head was maintained perpendicular to the skin surface, and the endpoint of the treatment was considered to be mild erythema, followed by ice application for 20 min. The treatment was performed once every four weeks for 12 weeks. In the combination therapy group, patients were treated with IPL therapy and microneedling therapy alternating at two‐week intervals for the same 12‐week period. Microneedle therapy was performed as follows: Facial anesthesia was first administered. The microneedle was then inserted perpendicularly into the skin, with unordered scrolling maneuvers performed until diffuse erythema was achieved as the treatment endpoint. Immediately after the microneedling procedure, EVE‐CHARM whitening lotion was applied specifically to the areas affected by melasma pigmentation and left to absorb completely for 3–5 min. A sterile healing‐promoting mask, formulated with sodium hyaluronate and recombinant type III collagen, was subsequently applied on the face for 40 min to minimize redness. Patients were instructed not to wet their faces for 12 h after treatment, and were protected from sunlight after every treatment.

### 2.5 Assessment

Document essential information for each patient, including their age, gender, and skin type classification. During each appointment, standardized facial photographs were captured using the VISIA analysis system and a high‐resolution camera. At baseline, and Weeks 4, 8, and 12, experienced dermatologists assessed the effectiveness of the treatment regimen in improving melasma by using the Modified Melasma Area and Severity Index (mMASI) Scores, which were informed by factors including area coverage and color intensity. The therapeutic effect was evaluated by the Symptom Score Reduction Index (SSRI) = [(baseline score − post‐treatment score)/baseline score] × 100%, with efficacy categorization: 0% = null, 1%–25% = mild, 26%–50% = moderate, 51%–75% = significant, 76%–100% = excellent. Treatment adverse events underwent protocol‐defined documentation throughout the study duration. Patient satisfaction regarding the final treatment outcome was assessed utilizing a 5‐category scale.

### 2.6 Statistical Analysis

Data statistical analysis and visualization were conducted in GraphPad Prism version 8.0. Pre‐post comparisons utilized Two‐way ANOVA (or mixed model) or T‐test, where *p* < 0.05 defined statistical significance. Results were presented as mean ± SEM.

## 3 Results

### 3.1 Patients' Characteristics

Thirty female subjects aged 40.5 ± 5.25 years (range 33–53 years), presenting with mixed melasma, classified as Fitzpatrick phototypes III to IV, were recruited in this study. Among these patients, twelve exhibited the central facial type of melasma, while eighteen exhibited the zygomatic type, and no visible blood vessels were observed in any of the patients.

### 3.2 Efficacy

**FIGURE 1:**
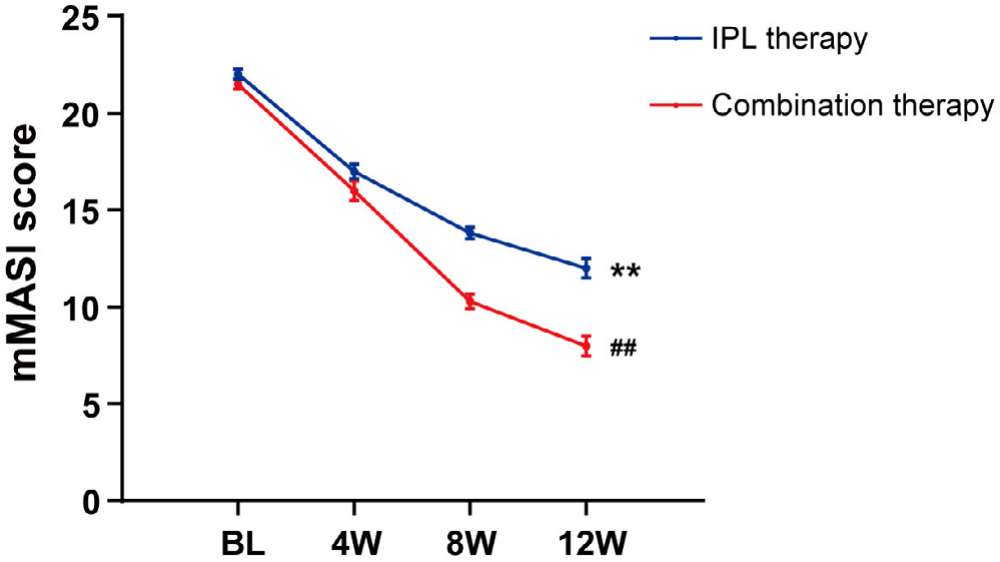
Mean mMASI score of the IPL therapy group and the combination therapy group was evaluated from baseline to the final visit. **p < 0.01 compared with BL. ^##^ *p* < 0.01 compared with the IPL therapy group. BL, baseline; 4 W, week 4; 8 W, week 8; 12 W, week 12.

**FIGURE 2:**
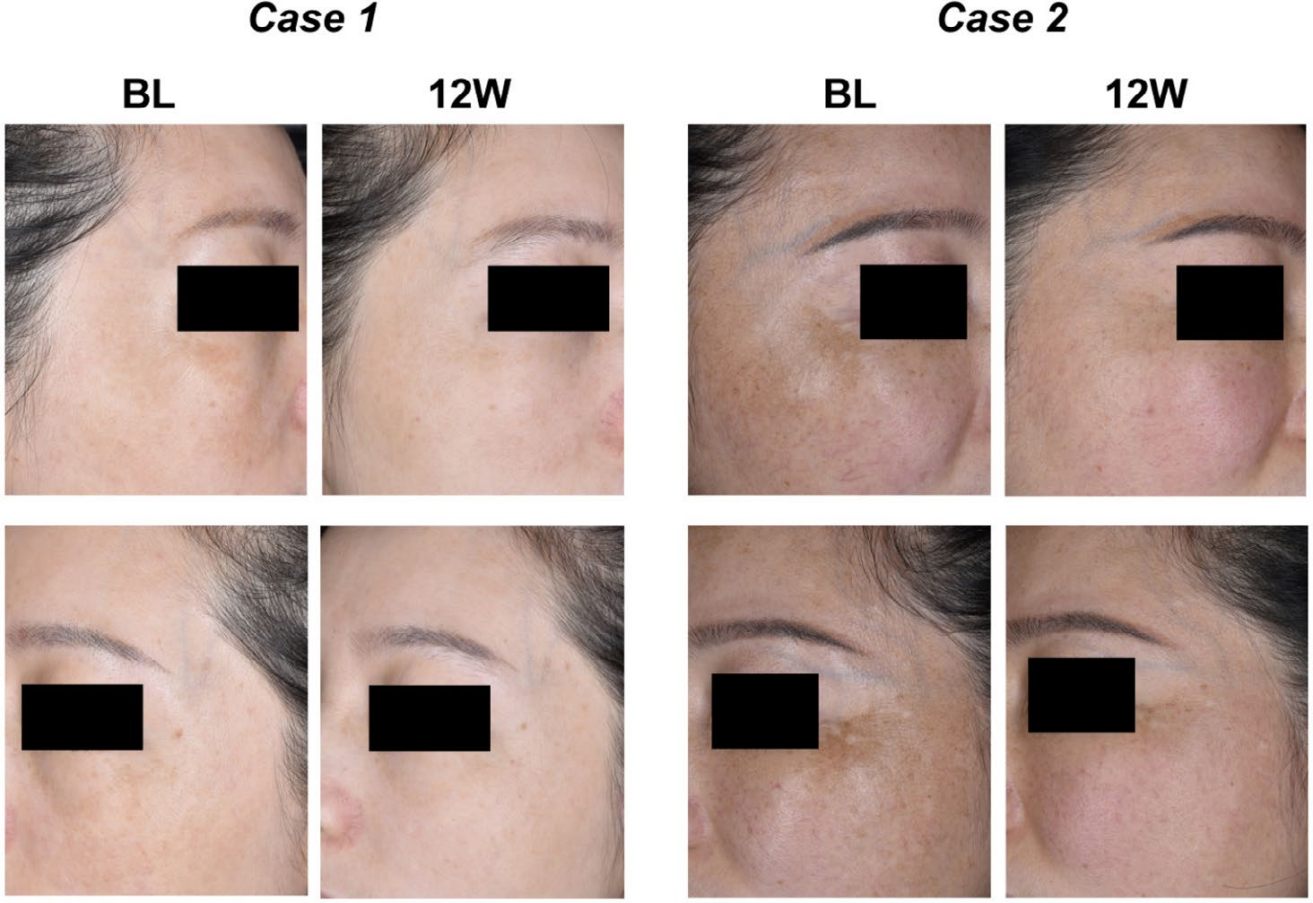
Clinical imaging records of representative cases in the combination therapy group at the baseline and week 12. BL, baseline; 12 W, week 12.

Medical professionals employ the mMASI score to evaluate the improvement of a patient's melasma, which is an internationally recognized method of assessing the severity of melasma. Quantification of the area and depth of pigmentation associated with melasma is accommodated, and the formula is as follows: The mMASI score = 0.3 forehead (A) (D) +0.3 right malar (A) (D) +0.3 left malar (A) (D) +0.1 chin (A) (D). The coefficients of 0.3, 0.3, 0.3, and 0.1 represent the respective proportions of the total facial area. A represents the area, which is divided into different grades from 0 to 6: absent, < 10%, 10%–29%, 30%–49%, 50%–69%, 70%–89% and 90%–100%. D stands for darkness, divided into different degrees from 0 to 4: absent, slight, mild, marked and severe. Compared with the baseline, individuals exhibited a statistically significant reduction in the mMASI score following the administration of IPL therapy or combination therapy (Figure 1). Furthermore, the statistical analysis of the mMASI scores at baseline revealed no significant differences between participants in the IPL therapy group and the combination therapy group, ensuring the comparability of the data, and we found that the combination therapy group exhibited a greater improvement in treatment outcomes (Table 2). The recorded mMASI scores were calculated using the SSRI formula to objectively assess the treatment effect. Of the total fifteen patients in the combination therapy group, thirteen demonstrated excellent improvement, while one patient each exhibited significant and moderate improvement. Figure 2 presents two representative examples from the combination therapy group. Following a 12‐week regimen of combined treatment, the patient showed a reduction in facial speckle, a decrease in inflammation, and an improvement in hyperpigmentation. The overall efficacy of the treatment achieved the anticipated outcomes.

**TABLE 2:** The mean mMASI score of the IPL therapy group and the combination therapy group.

	Baseline	Week 4	Week 8	Week 12
IPL therapy group	22.02 ± 1.00	16.99 ± 1.49	13.83 ± 1.15	12 ± 2.00
Combination therapy group	21.50 ± 0.99	15.99 ± 2.00	10.29 ± 1.51	7.99 ± 2.00

### 3.3 Objective Assessment

**FIGURE 3:**
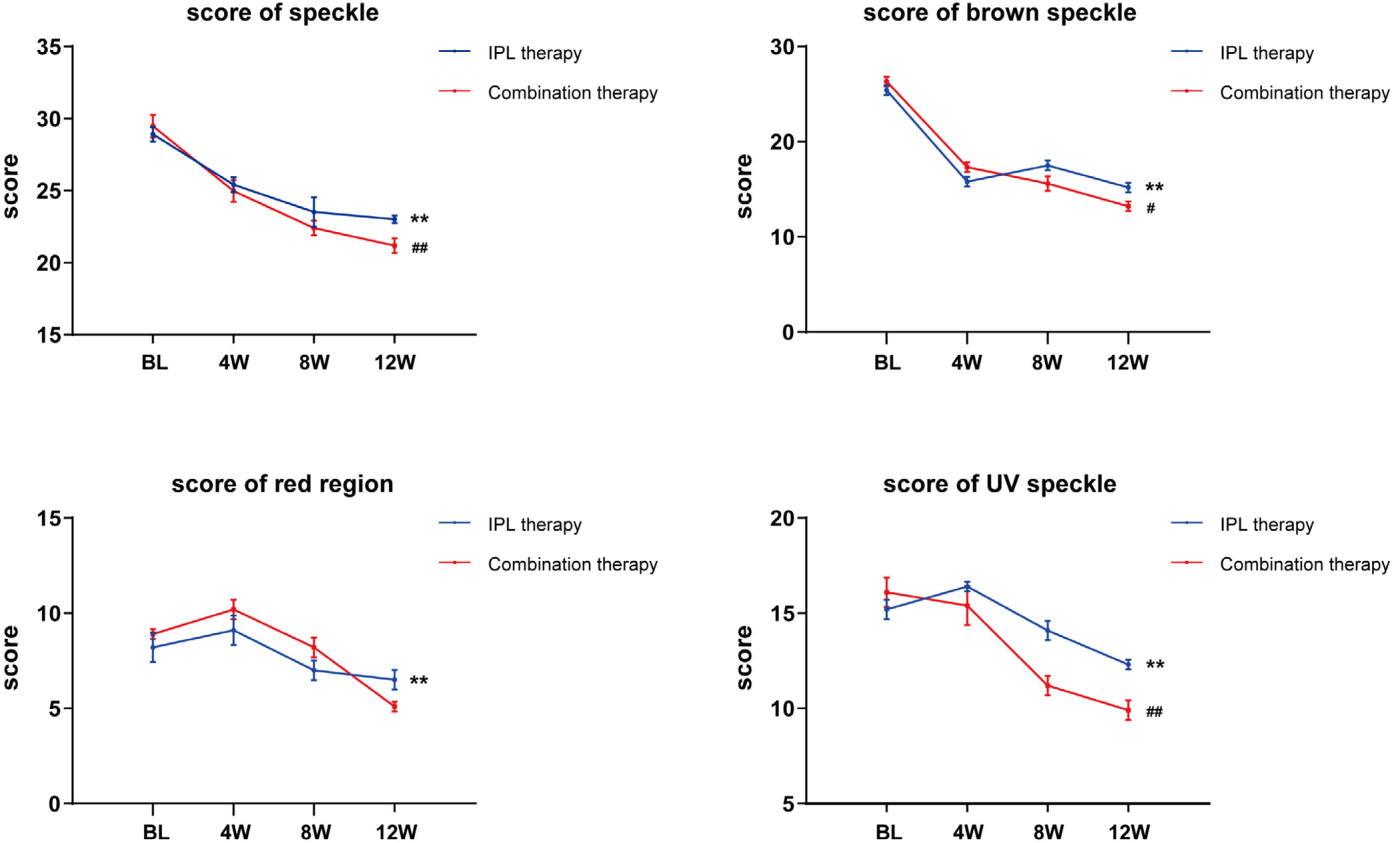
Mean score of speckle, brown speckle, red region and UV speckle recorded by VISIA platform for the IPL therapy group versus the combination therapy group. **p < 0.01 compared with BL. ^#^ *p* < 0.05, ^##^ *p* < 0.01 compared with the IPL therapy group. BL, baseline; 4 W, week 4; 8 W, week 8; 12 W, week 12.

VISIA skin analysis (Canfield Scientific, USA) has been validated in previous studies as a reliable and reproducible tool for assessing pigmentation disorders, including melasma, and has been widely used in clinical trials to provide objective outcome measures [22]. According to the established timeline, we used the VISIA platform to obtain standardized digital photographs of patients and systematically recorded a series of quantitative parameters, including scores for speckle, brown speckle, red region and UV speckle. The lower the score, the less severe the skin problems in that dimension, and the better the skin condition; conversely, the higher the score, the more prominent the corresponding skin problems. As shown in Figure 3, the scores of speckle and brown speckle continued to decrease with increasing numbers of combined therapy sessions, indicating that the improvement in pigmentation effects gradually increased with the number of treatments, showing a clear positive trend, and this trend was superior to that of the IPL monotherapy group. Interestingly, compared with baseline, the scores of red region and UV speckle both decreased in the combination therapy group. These results suggest that the combination therapy has the potential to reduce inflammation and enhance the ability to resist ultraviolet radiation of the skin, helping to block UV‐induced post‐inflammatory hyperpigmentation and prevent further aggravation of melasma.

### 3.4 Patients' Subjective Assessment of Efficacy

**FIGURE 4:**
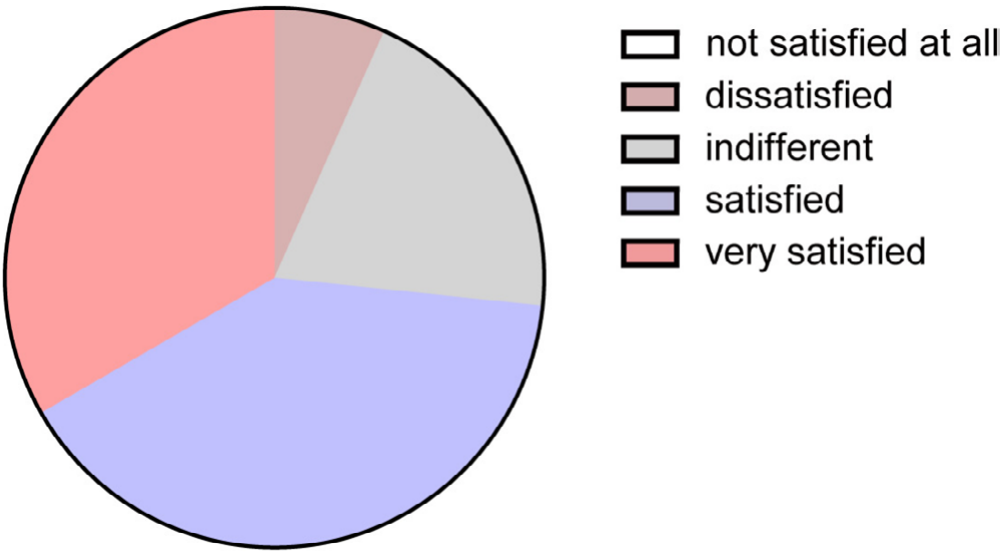
Proportion of patient satisfaction levels of the combination therapy group at the conclusion of the twelve‐week period.

Upon completion of the study, all participants were invited to provide feedback regarding their level of satisfaction with the final results. The findings showed that out of the 15 patients who received the combination treatment, 11 expressed very satisfied or satisfied, 3 were indifferent, and 1 was dissatisfied with the results. (Figure 4). In summary, a significant majority of participants, exceeding 73%, expressed satisfaction or a high level of satisfaction regarding the efficacy outcomes associated with the combination therapy for melasma. This observation is consistent with findings from prior efficacy outcome assessments.

### 3.5 Adverse Reactions

In the course of this study, all participants who received IPL monotherapy or combination therapy exhibited mild redness and swelling on the skin surface following the procedures, and patients in the IPL therapy group reported a more significant perception of pain and burning sensations during and subsequent to the treatment, but this level of discomfort was generally tolerated. Postoperative follow‐up indicated that the majority of patients recovered well from skin erythema within one week after the procedure, with no other serious adverse effects reported. It was essential to highlight that the patients who received combination therapy experienced a considerably lower incidence of adverse reactions when compared to those undergoing IPL therapy, especially the occurrence of post‐treatment hyperpigmentation, and the recovery period for the skin following the procedure was also notably shortened.

## 4 Discussion

Melasma is a challenging acquired hyperpigmented skin disorder with a prevalence of up to 30% in Asian women of childbearing age. In recent years, numerous studies have revealed the development of melasma is related to a series of biological processes, including overactivity of melanocytes, increased melanin synthesis and impaired transport, damage to the basement membrane bands, inflammatory responses, and photoaging [23, 24]. Notably, transepidermal water loss (TEWL) levels were significantly elevated in the lesion areas of melasma patients [25]. In addition, the expression of mediators associated with pro‐melanin synthesis, including microphthalmia‐associated transcription factor, tyrosinase (TYR) and tyrosinase‐related protein 1 (TYRP1), was also up‐regulated, the number of melanocytes increased, and the process of melanin synthesis became more active [26]. The basement membrane zone (BMZ) is an important structure located between the epidermis and the dermis, and is responsible for maintaining the exchange of substances and signaling functions between the two [27]. UV radiation may be one of the contributors that damage the basement membrane bands. The mechanism involves stimulating keratinocytes to generate elevated levels of reactive oxygen species and pro‐inflammatory mediators, which trigger oxidative stress and inflammation within the skin. These processes subsequently promote the secretion of matrix metalloproteinases (MMPs), leading to extracellular matrix degradation [28]. A histochemical and immunohistochemical study found that more than 80% of patients with melasma showed signs of damage to the basement membrane bands in the skin surrounding the lesions [29]. This damage may cause active melanocytes and melanin granules to migrate from the epidermis to the dermis, triggering persistent dermal hyperpigmentation in melasma [30]. In summary, based on the fact that melasma exists in the epidermis, BMZ and dermis with tissue structural changes, rather than just a pure pigmentation problem, the management strategy for melasma tends to be a combination of multiple treatments, with the guiding principles of reducing melanin production, anti‐inflammation, repairing the skin barrier, and counteracting photoaging [31].

IPL is an effective intervention for melasma, which selectively targets melanin and hemoglobin in the melasma lesion area, utilizing a thermal effect to disrupt melanosomes and facilitate their decomposition, while coagulating abnormal blood vessels to reduce the inflammatory response [32]. Furthermore, IPL therapy enhances cutaneous microcirculation and stimulates fibroblast activity, promoting the synthesis of collagen and elastin fibers, which helps to remodel the tight structure of the skin [33]. Although IPL monotherapy has shown significant improvement in epidermal pigmentation, the effect on dermal melanin is relatively limited. There is even a potential risk of damaging the BMZ, as well as the possibility of inducing melanocyte overactivation and migration [34].

As an auxiliary method of localized chloasma treatment, microneedling technology promotes the deep penetration of drugs into the epidermis or dermis by establishing micro‐channels on the skin surface, thus enhancing the absorption efficiency of the drugs while reducing the degree of damage to the skin, shorter recovery time allows most patients to return to their normal work and life within 1–2 days after the treatment [14, 35]. Delivering whitening formulas such as tranexamic acid and vitamin C in this way can effectively inhibit the activity of TYR in melanocytes and significantly reduce melanin synthesis [36]. In addition, studies have revealed that microneedling can treat melasma by improving the skin's photoaging pattern, with improved sunlight elastic tissue proliferation, increased epidermal thickness, and restoration of collagen in the basement membrane bands and superficial dermis [37, 38]. Therefore, it is feasible to combine microneedling with IPL for chloasma treatment, which can make up for the shortcomings of IPL monotherapy and further improve efficacy.

The skin microbiota is integral to preserving the integrity of the skin barrier function, and many skin diseases are associated with skin microecological imbalance [39, 40]. UV irradiation and photoaging have been shown to disturb microbial composition, with a marked reduction in beneficial genera such as Lactobacillus. This dysbiosis is associated with decreased levels of antioxidant molecules, increased collagen degradation, and elevated expression of inflammatory factors [41]. Postbiotics, which consist of non‐viable bacterial components or metabolites, have recently attracted increasing attention for their therapeutic potential in skin health initiatives. Notably, various strains and their metabolites exhibit distinct mechanisms of action in regulating skin health. Numerous studies have demonstrated the positive impact of Lactobacillus and its fermentation derivatives on skin health. Our previous research has demonstrated that microneedling in combination with postbiotic preparation (Lactobacillus fermentation lysate serves as the primary component) is highly effective in improving acne lesions, alleviating inflammation, and strengthening the skin barrier function [42]. The cell‐free supernatant derived from *Lactobacillus gasseri* BNR17 demonstrated an inhibitory effect on melanin production in human keratinocytes [43]. This behavior is associated with a reduction in tyrosinase activity, downregulation of melanogenesis‐related gene expression, and an upregulation of the HO‐1 antioxidant gene expression. Tyndallized *Lactobacillus acidophilus* KCCM12625P (AL) inhibited melanogenesis by regulating the cAMP signaling pathway and significantly down‐regulating the mRNA expression of TYR and TYRP‐1 [44]. In addition, lactic acid bacteria lysate accelerates the epithelialization process by promoting keratinocyte migration, reduces pro‐inflammatory mediator levels, and promotes wound healing by regulating specific signaling pathways (such as NF‐κB, Hedgehog, and RUNX signaling pathways) [45]. These evidences reveal that Lactobacillus fermentation lysate has multiple potential effects: (1) Regulates inflammatory and antioxidant pathways to mitigate skin inflammation. (2) Maintains a healthy skin microbiome by suppressing pathogenic bacteria and supporting the growth of beneficial microorganisms, thereby strengthening the skin barrier function. (3) Promotes epithelial cell metabolism and accelerates skin renewal. (4) Improves pigmentation by modulating melanin synthesis pathways. Consequently, owing to their numerous advantages and favorable safety profile, Lactobacillus fermentation lysate products are increasingly recognized as valuable ingredients in the formulation of innovative skincare solutions, particularly showing great potential in the management of pigmentary conditions, including melasma.

This study was the first to investigate the efficacy of IPL monotherapy versus the combination treatment of IPL and microneedling with EVE‐CHARM whitening lotion for melasma. We observed a statistically significant decrease in mMASI scores following the administration of the second combination treatment at week 8, in comparison to the results obtained from the IPL monotherapy group. After three combined treatments, lesions, pigmentation, inflammation, and photoaging symptoms were all improved, possibly due to the synergistic effects of IPL and microneedling, which together promote melanin destruction and metabolism, as well as collagen regeneration. On the other hand, microneedling may contribute to the repair of the skin barrier and enhance the inhibition of melanin synthesis by creating microchannels to deliver the EVE‐CHARM whitening lotion, which is enriched with Lactobacillus fermentation lysate, niacinamide and tranexamic acid. Thus, the combination of IPL and microneedling realizes synergistic effects by repairing the skin barrier, inhibiting melanin production, improving skin texture, and reducing the incidence of adverse reactions, which provides a safe and efficacious approach to addressing facial melasma. This study has certain limitations: (1) The sample size is still relatively small. (2) The participant cohort consisted exclusively of female subjects. (3) The duration of the follow‐up period was comparatively brief. In the future, extensive clinical trials should be conducted to comprehensively assess its long‐term outcomes and broader applicability.

## 5 Conclusion

This study investigated the therapeutic synergy of the combination of IPL and microneedling with a postbiotic formulation for facial melasma. Based on the photothermal effect of IPL and the mechanical stimulation of microneedles, the combination of the two facilitates the breakdown and metabolism of melanin, while simultaneously promoting epidermal renewal and the regeneration of collagen. What's more, the application of EVE‐CHARM whitening lotion following microneedling not only mitigates inflammation and melanin production, but also fosters the equilibrium of the skin's microflora and aids in the restoration of the skin barrier that effectively contributes to a reduction in the recovery time of the skin post‐procedure. Consequently, the combination therapy of IPL with microneedling, alongside the application of EVE‐CHARM whitening lotion shows promise as a multidimensional approach for melasma management and warrants validation in larger, controlled studies.

## Author Contributions

Conceptualization: Z.L. and Q.M. Methodology: J.M., Hu.W., Ha.W., and T.L. Validation: T.L., H.L., and J.H. Formal analysis: Z.L., G.H., L.C., J.W., and L.Y. Investigation: Y.X., J.M., Hu.W., Y.B., X.J., T.W., Y.Z., X.X., X.M., M.Y., and X.Z. Data curation: Y.X. and C.L. Writing – original draft preparation: Z.L. and Y.X. Writing – review and editing: Z.L., J.M., Ha.W., and Q.M. Visualization: Z.L. and H.Z. Supervision: H.Z., Y.C., M.O., and Q.M.

## Ethics Statement

Ethical approval was granted by the ethics committee of Guangzhou MLT Medical Cosmetic Clinic (No. 2024001). All recruited subjects were aware of the study process and signed the written consent forms.

## Consent

For all personal information used in this article, for example, personal images, consent for publication has been obtained.

## Conflicts of Interest

The authors declare no conflicts of interest.

## Supporting information


**Data S1:** jocd70580‐sup‐0001‐DataS1.docx.

## Data Availability

The data that support the findings of this study are available from the corresponding author upon reasonable request.
