# Supplementary material for: Efficacy of the Combination Therapy of Intense Pulsed Light and Microneedling With a Postbiotic Formulation for Melasma
Source: J Cosmet Dermatol. 2025 Dec 5;24(12):e70580. doi: 10.1111/jocd.70580 (PMC12679679; doi:10.1111/jocd.70580)
Supplement: Supplementary file 1 — Data S1: jocd70580‐sup‐0001‐DataS1.docx. [file JOCD-24-e70580-s001.docx]

**Supplementary material**

List of ingredients in EVE-CHARM® whitening lotion.

|  | **The lyophilized powder** | **The matched solution** |
| --- | --- | --- |
| Main ingredients | Water, mannitol, Lactobacillus fermentation lysate, trehalose, nicotinamide, tranexamic acid | Water, glycerol, 1,2-hexanediol, p-hydroxyacetophenone, allantoin, PEG-40 hydrogenated castor oil, panthenol, sodium hyaluronate |
| Trace ingredients | 1,2-pentanediol, carnosine, natriuretic peptide-1, p-hydroxyacetophenone, glycol, glycerol, hydrogenated lecithin ceramide NP, glycine, alanine, proline | Glycyrrhiza glabra root extract |

Stability assessment of EVE-CHARM^®^ whitening lotion.

| **Items** | **Test condition** | **Test sample** | **One-month results** |
| --- | --- | --- | --- |
| Light | The test materials were placed in a light box with fluorescent lamps and monitored once a week. | Five bottles each of the lyophilized powder and the accompanying solution | The product exhibits no discoloration, emits no odor, contains no impurities, and the packaging is securely sealed. |
| Cold | The test materials were placed in the refrigerator (-8 ~ -10℃) and monitored once a week. | Five bottles each of the lyophilized powder and the accompanying solution | The product exhibits no discoloration, emits no odor, contains no impurities, and the packaging is securely sealed. |
| Heat | The test materials were placed in a thermostat (48 ~ 50℃) and monitored once a week. | Five bottles each of the lyophilized powder and the accompanying solution | The product exhibits no discoloration, emits no odor, contains no impurities, and the packaging is securely sealed. |
| High-low temperature | The test materials were placed in the high-low temperature test chamber (-10 ~ -48℃) and monitored once a week. | Five bottles each of the lyophilized powder and the accompanying solution | The product exhibits no discoloration, emits no odor, contains no impurities, and the packaging is securely sealed. |
| Ambient | The test materials were placed at normal room temperature and monitored once a week. | Five bottles each of the lyophilized powder and the accompanying solution | The product exhibits no discoloration, emits no odor, contains no impurities, and the packaging is securely sealed. |
